# Quality of life after rectal cancer surgery: differences between laparoscopic and transanal total mesorectal excision

**DOI:** 10.1007/s00464-018-6276-z

**Published:** 2018-07-02

**Authors:** Marloes Veltcamp Helbach, Thomas W. A. Koedam, Joep J. Knol, Simone Velthuis, H. Jaap Bonjer, Jurriaan B. Tuynman, Colin Sietses

**Affiliations:** 1 Department of Surgery, Gelderse Vallei Hospital, Willy Brandtlaan 10, 6716 RP Ede, The Netherlands; 2 Department of Surgery, VU University Medical Centre, De Boelelaan 1117, 1081 HV Amsterdam, The Netherlands; 3 Department of Surgery, Jessa Hospital, Salvatorstraat 20, 3500 Hasselt, Belgium

**Keywords:** Transanal TME, TAMIS, Rectal cancer, Surgery, Quality of life

## Abstract

**Background:**

Transanal total mesorectal excision (TaTME) is a safe alternative to laparoscopic TME for mid and low rectal cancer. TaTME allows improved visualization of the surgical planes and margins, and may potentially improve oncological outcomes. However, functional results after total mesorectal excision (TME) are variable and there are currently only a few published studies that include functional data related to the outcomes of TaTME.

**Methods:**

Fifty-four consecutive patients were included in this study: one group included 27 patients who underwent laparoscopic low anterior and the other included 27 patients who underwent TaTME. All patients were asked to complete five questionnaires related to quality of life (QOL) and function [EQ-5D-3L, EORTC-QLQ C30, EORTC-QLQ C29, Low Anterior Resection Syndrome score (LARS), and International Prostate Symptom Score IPSS]. All TaTME patients were operated on at The Gelderse Vallei Hospital by a single surgeon and had a follow-up of at least 6.6 months.

**Results:**

The EORTC-QLQ C30 and EQ-5D-3L questionnaires showed comparable outcomes in terms of QOL between the two groups. Almost all items evaluated by the EORTC-QLQ C29, including sexual outcomes, were similar between the two groups. One item concerning fecal incontinence, however, was scored worse for TaTME. There were no significant differences between the groups in terms of LARS symptoms or urinary function.

**Conclusions:**

Patients undergoing laparoscopic or transanal TME showed comparable functional and QOL outcomes. Although the TaTME technique is still evolving, this study indicates that this technique is a safe alternative to laparoscopic surgery in terms of functional outcomes for mid and low rectal cancers.

Transanal total mesorectal excision (TaTME) is a relatively new surgical technique that is expected to improve rectal cancer care. During TaTME, rectal resection is performed transanally thereby improving visualization of the most difficult portion of the dissection [1, 2]. Improved visualization may prevent injury to the hypogastric plexus and potentially improve outcomes. Specimen quality, important for quality assurance, has been consistently good following TaTME. In addition, potential short-term clinical advantages, such as lower conversion rate, lower leak rate, and slightly lower short-term morbidity, have been reported [3, 4]. Transanal endoscopic mobilization allows very low dissection under direct vision. Due to improved visualization of the margins and surgical planes, patients with low rectal tumors, who would have otherwise been excluded from dissection without abdominoperineal resection (APR), now have the option of a low coloanal anastomosis. Despite these positive outcomes, some features of the TaTME procedure are still poorly understood. As such, there is concern that TaTME may impede good functional outcomes due to the relatively low anastomosis or by damage to the internal sphincter [5, 6].

Functional outcomes after TME are highly variable [7]. Long-term results reported from the Dutch TME trial indicate that a large proportion of patients have experienced poor functional outcomes after TME [8]. Outcomes are influenced by tumor height, neoadjuvant therapy, and the type of anastomosis [5, 6, 9]. In addition, the type of surgery employed and the experience of the surgical team have a major impact on outcome. The OSTRiCh (Consortium for Optimizing the Treatment of Rectal Cancer) group [10] showed that an experienced surgical team could potentially avoid APR and instead create a low anastomosis, thereby avoiding a permanent colostomy. Positioning of the transanal platform into the anal canal could also potentially damage the sphincter muscle and negatively influence functional outcomes [11].

This study aimed to report short-to-medium term functional outcomes in patients who underwent TaTME surgery for rectal cancer compared to patients who underwent rectal cancer surgery prior to introduction of TaTME.

## Materials and methods

### Study design

This study describes the functional results of two cohorts of patients who underwent TME for rectal cancer. The first group included patients who underwent TME between January 2010 and June 2012, using a standard laparoscopic low anterior resection (LAR group) approach and the second group included patients who underwent TaTME after its introduction in March 2012, using a transanal approach (TaTME group).

Patients with tumors up to 15 cm from the anal verge who were treated by TME, either laparoscopically or transanally, with primary anastomosis and without a current stoma were included. All of the transanal procedures were performed by a single surgeon (CS), while three attending surgeons performed the laparoscopic procedures.

After Institutional Review Board approval, all patients were sent questionnaires relative to quality of life and functional results at least 6 months (range 6.6–78.0) after stoma reversal.

### Preoperative staging and neoadjuvant treatment

All patients were treated according to the Dutch protocols for rectal cancer treatment [12]. Depending on the tumor grade, patients received neoadjuvant radiotherapy or chemoradiotherapy or surgery alone. No patients had adjuvant chemotherapy as this is not indicated by the Dutch protocols. Patients received preoperative mechanical bowel preparation with Moviprep (Norgine, Amsterdam, The Netherlands). All patients initially received epidural analgesia, followed by patient-controlled analgesia for postoperative pain control and prophylactic antibiotics were administered according to protocol. Postoperatively, all patients were treated according to the enhanced recovery after surgery (ERAS) guidelines [13].

### Surgical procedure

#### Laparoscopic TME

A traditional four-trocar medial-to-lateral technique was employed, as previously described. All specimens were extracted through an umbilical incision after placement of a wound protector. In all cases, a side-to-end anastomosis was created using a 31 EEA stapler (Covidien, Mansfield, Massachusetts, USA) with a temporary diverting loop ileostomy.

#### Transanal TME

The transanal approach was performed as previously described [1, 2]. A standard approach was used, beginning with transanal mobilization of the low and mid rectum and splenic flexure mobilization. The sigmoid and upper rectum were mobilized during the laparoscopic approach.

In the first group of patients, the specimen was transanally extracted, while in subsequent patients it was removed through the ileostomy site. The end-to-end anastomosis in the first group and the side-to-end anastomosis in the second group were created using a 33 EEA hemorrhoid stapler (Covidien, Mansfield, Massachusetts, USA).

### Stoma reversal

Approximately 6 weeks after surgery, the temporary stoma was reversed, after sigmoidoscopic visualization confirmed the integrity of the anastomosis.

### Functional assessment

The questionnaires included instruments that measured quality of life and anorectal function.

#### EQ-5D-3L

Evaluates the level of mobility, self-care, activity, pain, and anxiety, using two different scoring systems. The EQ-5D index generates an overall score and the EQ-VAS assesses global health status on a visual analogue scale, ranging from 0 (worst) to 100 (best).

#### EORTC-QLQ C30

Measures the quality of life (QoL) of cancer patients using three scales: a functional scale (5 items), a symptom scale (9 items), and a global health status/quality of life. Individual scores were analyzed according to the EORTC scoring manual and converted to a score ranging from 0 to 100. A higher score on the symptom scale indicates a worse health-related quality of life. On the global health status and functional scales, a higher score indicates better health-related quality of life [14, 15].

#### EORTC-QLQ C29

Supplementary module designed to complement the QLQ-C30, consisting of 29 items including items in 4 scales (urinary frequency, blood/mucus in stools, stool frequency, body image) and 19 single items. Individual scores were converted to a score ranging from 0 to 100. In the symptom scale, a higher score indicates worse health-related quality of life and on the functional scale a higher score indicates better health-related quality of life [16].

#### LARS (Low anterior resection syndrome)

Five questions designed for quick evaluation of ano(neo)rectal and bowel function after rectal cancer surgery, including flatus incontinence, liquid stool incontinence, bowel frequency, clustering of stools, and urgency. Total score indicates one of three degrees of LARS: no LARS (0–20), minor LARS (21–29), or major LARS (30–42) [17].

#### IPSS (International Prostate Syndrome Score)

Measures quality of urinary function in male subjects with seven questions that can be answered on a scale from ‘never’ (score 0) to ‘almost always’ (score 5). Using the total score, the quality of urinary function is graded as mild (0–7), moderate (8–19), or severe (20–35) [18].

### Statistical analysis

Data are presented as means or categories, with *p* values determined by Students *t* test and Chi-Square test or, if not applicable, using the Mann–Whitney *U* test or Fisher–Freeman–Halton test.

## Results

### Baseline characteristics

Sixty-four patients were eligible for inclusion in the study and were sent the questionnaires. Of these, 10 failed to respond for a response rate of 84.3%. The 54 patients LAR: 27, 74% males; TaTME: 27, 67% males who did complete all 5 of the questionnaires were included in the study (Table 1). Although there was no significant difference noted between the groups in terms of gender (74 and 27%, LAR and TaTME, respectively), a significant difference was observed in the mean age (62.7 and 68.0 *p* = 0.04). In addition, a significant difference was noted in the mean follow-up (59.5 vs. 20.0 months, respectively). Body mass index (BMI), ASA (American Society of Anaesthesiologists) classification, tumor height, and neoadjuvant therapy did not significantly differ between the groups (Table 1).

**Table 1:** Baseline characteristics

	LAR (*n* = 27)	TaTME (*n* = 27)	*p* value
Age (mean, 95% CI)	62.7 (59.6–65.7)	68.0 (64.4–71.6)	.040
Sex			
Male	20	18	.766
Female	7	9	
BMI (mean, 95% CI)	26.1 (25.1–27.3)	27.6 (25.7–29.5)	.364
ASA			
I	13	5	.062*
II	12	20	
III	2	2	
Tumor height^a^			
Low	7	9	.569*
Mid	18	14	
High	2	4	
Type of anastomosis			
Side-to-end	27	4	.000
End-to-end	0	23	
Temporary diverting stoma	22	22	1.000
Neoadjuvant therapy			
None	5	9	.395*
RT	18	16	
CRT	4	2	
Follow-up questionnaire (months) (median)	59.5	20.0	.000*
Range	39.7–82.0	6.6–44.4	
Postoperative complications (CD)			
IIIa–V	7	3	.161
Pathology stage			
T0–1	6	4	.647
T2	9	12	
T3	12	11	
Completeness mesorectum			
Incomplete	0	0	.236*
Nearly complete	2	0	
Complete	24	27	
CRM involvement			
No	27	27	1.000
Yes	0	0	

*LAR* low anterior resection, *TaTME* transanal total mesorectal excision, *BMI* body mass index (kg/m^2^), *RT* radiotherapy, *CRT* chemoradiotherapy, *CD* Clavien–Dindo classification

*Calculated by Fisher–Freeman–Halton test instead of Chi-Square test or Mann–Whitney *U* test instead of Student’s *t* test

^a^Tumor height: cm from anal verge on MRI, Low = 0–5 cm, Mid = 6–10 cm, High = 11–15 cm

Twenty-two patients in each group had a temporary diverting ileostomy (Table 1). A significant difference between the groups in terms of the type of anastomosis was noted. In the laparoscopic group, all anastomoses were created ‘side-to-end.’ In the TaTME group, we started with performing an end-to-end anastomosis and changed this to side-to-end anastomosis after changing the extraction site (23 and 4 patients, respectively).

In terms of pathology, no differences were seen between the groups relative to stage or outcomes (Table 1). The mesorectum was reported as complete for all 27 patients in the TaTME group. A nearly complete mesorectum was noted in two patients in the laparoscopic group, while the remaining 25 were reported as complete. No involvement of the circumferential resection margin (CRM) or recurrence was seen in any patients. In addition, no differences were noted in terms of postoperative morbidity and 90-day complications, between the groups.

### Questionnaire results

#### Quality of life

##### EQ-5D-3L

Quality of life measures did not reveal any significant differences (Table 2). Overall health status was graded as 79.1 and 75.6 for the LAR and TaTME groups, respectively (*p* = 0.400). An overall summary as well as individual analysis of the five questions regarding mobility, self-care, activity, pain, and anxiety were comparable (*p* = 0.159) between the groups.

**Table 2:** EQ-5D-3L

	LAR (*n* = 27)	TaTME (*n* = 27)	*p* value
EQ-5D VAS (mean, 95% CI)	79.1 (72.8–85.3)	75.6 (69.9–81.3)	.400
EQ-5D index (mean, 95% CI)	92.8 (88.2–97.4)	88.1 (83.1–93.1)	.159
Mobility			.340
Level I	22	19	
Level II	5	8	
Level III	0	0	
Self-care			1000*
Level I	25	26	
Level II	2	1	
Level III	0	0	
Activity			.260*
Level I	22	18	
Level II	4	8	
Level III	0	1	
Pain/discomfort			.535
Level I	21	19	
Level II	6	8	
Level III	0	0	
Anxiety/depression			.704*
Level I	24	22	
Level II	3	5	
Level III	0	0	

*EQ-5D-3L* euroquol group five dimensions three levels, *VAS* visual analogue scale, *Level I* indicating no problem, *Level II* indicating some problems, *Level III* indicating extreme problems

*Calculated by Fisher–Freeman–Halton test instead of Chi-Square test

##### EORTC QLQ-C30

Only questions regarding financial difficulties, role functioning, and fatigue were found to be significantly different, favoring the LAR group (*p* = 0.032, *p* = 0.042 and *p* = 0.021, respectively) (Table 3). The colorectal-focused questions concerning diarrhea and constipation were comparable between the two groups.

**Table 3:** EORTC QLQ-C30

Symptom^a^	LAR	TaTME	*p* value
	Mean (*n*)	Mean (*n*)	
Fatigue	14.0 (27)	26.5 (26)	.021
Nausea and vomiting	2.5 (27)	3.1 (27)	.987
Pain	3.7 (27)	12.8 (26)	.051
Dyspnea	9.9 (27)	23.5 (27)	.214
Insomnia	14.8 (27)	18.0 (26)	.385
Appetite loss	2.50 (27)	7.4 (27)	.358
Constipation	9.9 (27)	8.6 (27)	.763
Diarrhea	3.7 (27)	16.0 (27)	.070
Financial difficulties	2.4 (27)	14.8 (27)	.032
Global health status^a^	83.6 (27)	79.6 (26)	.208
Functional^b^			
Physical functioning	88.1 (27)	83.2 (27)	.128
Role functioning	89.5 (27)	80.2 (27)	.042
Emotional functioning	90.1 (27)	89.4 (26)	.887
Cognitive functioning	90.1 (27)	89.4 (27)	.860
Social functioning	92.6 (27)	87.7 (27)	.093

Means calculated by Mann–Whitney *U* Test

*EORTC QLQ-C30* European Organization for Research and Treatment of Cancer Quality of Life Questionnaire Core, *LAR* low anterior resection, *TaTME* transanal total mesorectal excision score range 0–100

Score range 0–100

^a^Higher score indicates worse health-related quality of life

^b^Higher score indicates better health-related quality of life

##### EORTC QLQ-C29

Hair loss and sore skin were reportedly worse in the TaTME vs the LAR group (*p* = 0.10 and *p* = 0023, respectively) (Table 4). Moreover, fecal incontinence, which was scored by a single item, differed significantly between the two groups in disfavor of TaTME (33.3 vs. 16.7 in LAR and TaTME respectively; *p* = 0.032). Other questions related to stool frequency and mucus frequency were comparable between the groups. Similarly, sexual outcomes such as impotence in males and dyspareunia in females as well as sexual interest were similar between the two groups.

**Table 4:** EORTC QLQ-C29

Symptom^a^	LAR	TaTME	*p* value
	Mean (*n*)	Mean (*n*)	
Urinary frequency	28.4 (27)	38.9 (27)	.101
Blood and mucus	3.7 (27)	3.7 (27)	1.00
Stool frequency	30.7 (25)	36.5 (26)	.556
Urinary incontinence	9.9 (27)	7.4 (27)	.886
Dysuria	1.2 (27)	2.5 (27)	.556
Abdominal pain	7.4 (27)	10.3 (26)	.643
Buttock pain	12.3 (27)	24.7 (27)	.114
Bloating	14.8 (27)	14.8 (27)	1.00
Dry mouth	8.6 (27)	29.8 (27)	.156
Hairloss	0.0 (27)	9.9 (27)	.010
Taste	6.2 (27)	17.3 (27)	.083
Flatulence	39.7 (26)	41.0 (26)	.975
Fecal incontinence	16.7 (26)	33.3 (25)	.032
Sore skin	7.7 (26)	26.9 (26)	.023
Embarrassment	28.2 (26)	38.5 (26)	.180
Impotence (men)	51.0 (17)	41.0 (13)	.483
Dyspareunia (women)	8.3 (5)	7.4 (9)	.905
Functional^b^			
Body image	90.9 (27)	88.4 (25)	.325
Anxiety	75.3 (27)	74.4 (26)	.715
Weight	84.1 (26)	87.2 (26)	.493
Sexual interest (men)	63.3 (20)	68.9 (15)	.564
Sexual interest (women)	73.3 (5)	83.3 (6)	.662

*EORTC QLQ-C29* European Organization for Research and Treatment of Cancer Quality of Life Questionnaire Core, *LAR* low anterior resection, *TaTME* transanal total mesorectal excision

Score range 0–100

^a^Higher score indicates worse health-related quality of life

^b^Higher score indicates better health-related quality of life

#### Anorectal function

##### LARS

Although patients in both groups reported LARS, no significant difference in the severity was identified between the two groups (8 vs. 16 major LARS, respectively; *p* = 0.087). Furthermore, the mean LARS questionnaire scores were equivalent between the two groups (24.0 vs. 27.7, respectively; *p* = 0.131). When comparing the questionnaire items individually, including incontinence to flatus and liquid stool, no significant differences were seen between the groups (Table 5).

**Table 5:** LARS

	LAR (*n* = 27)	TaTME (*n* = 27)	*p* value
Incontinence for flatus			.829*
Never	2	2	
< Once a week	8	11	
≧ Once a week	17	14	
Incontinence for liquid stools			.056
Never	12	5	
< Once a week	10	10	
≧ Once a week	5	12	
Bowel frequency			.409*
1–3 times a day	13	14	
4–7 times a day	12	8	
> 7 times a day	0	2	
< Once a day	2	3	
Clustering of stools			.213
Never	5	6	
< Once a week	12	6	
≧ Once a week	10	15	
Urgency			.208
Never	11	6	
< Once a week	11	11	
≧ Once a week	5	10	
LARS categories			
No (*n*)	11	7	.087
Minor (*n*)	8	4	
Major (*n*)	8	16	
LARS (mean 95% CI)	24.0 (19.9–28.2)	27.7 (22.3–32.8)	.267

*LARS* low anterior resection syndrome, *No* score 0–20, *Minor* score 21–29, *Major* score 30–42, *LAR* low anterior resection, *TaTME* transanal total mesorectal excision

*Calculated by Fisher–Freeman–Halton test instead of Chi-Square test

#### Urinary function

##### IPSS (Male Patients)

No significant differences were seen when comparing IPSS scores per subgroup, (*p* = 0.277). Moreover, when analyzing mean questionnaire scores, comparable outcomes were found between the two surgical approaches (*p* = 0.512) (Table 6).

**Table 6:** IPSS

	LAR (*n* = 18)	TaTME (*n* = 14)	*p* value
Mild (*n*)	12	7	.277*
Moderate (*n*)	5	7	
Severe (*n*)	1	0	
IPSS (mean, 95% CI)	6.7 (3.6–9.9)	8 (4.2–11.8)	.582

*IPSS* international prostate syndrome score, *Mild* score 0–7, *Moderate* score 8–19, *Severe* score 20–35, *LAR* low anterior resection, *TaTME* transanal total mesorectal excision

*Calculated by Fisher–Freeman–Halton test

## Discussion

The short-to-medium term functional outcome data reported in our study, including anorectal function and quality of life, did not reveal any major differences between the transanal and laparoscopic TME groups. However, there were significant differences in quality of life favoring the laparoscopic approach to TME in terms of role functioning, fatigue, and financial difficulties. Role functioning, limitations in pursuing daily activities or leisure time activities, and fatigue can probably be attributed to differences in length of follow-up. Patients in the TaTME group had less postoperative recovery time, therefore worse outcome was reported. However, we could not attribute the difference in financial difficulties between the two groups to any specific cause.

The results of the EORTC QLQ-C29 questionnaire showed a significant difference between the two groups in terms of fecal incontinence scored by a single item regarding leakage of stools, favoring the LAR group. The questionnaire was mainly developed for quality of life evaluation, which includes some questions specific to function. Other outcomes regarding stool-related questions in this questionnaire and in the EORTC QLQ-C30 were similar between the groups. Differences in individual questions on function should therefore be addressed with caution, especially because no differences were seen in functional outcomes by the LARS questionnaire, which is developed specifically for anorectal function. Low Anterior Resection Syndrome was reported equally in both groups. Moreover, when analyzing the individual questions of the LARS questionnaire separately, including one regarding incontinence for liquid stool, again no significant differences were seen between the groups.

The functional outcomes reported in this study show that LARS symptoms occur with equal frequency in patients who underwent standard laparoscopy or those in whom TaTME was employed. These findings are comparable to reports in the literature. Long-term follow-up data from the Dutch TME trial showed that 46% of patients still endure major LARS symptoms as far out as 14 years after TME surgery [8]. A study by Pontallier et al. [19] reported severe symptoms after both transanal and laparoscopic approaches for low rectal cancer, with major LARS in 82 and 76% of the transanal and standard laparoscopy patients, respectively. A recently published study by Koedam et al. reported 33% major LARS in patients after TaTME [20]. While both studies showed significant LARS symptoms after transanal resection, their results cannot be compared because the two studies included different patient groups, with more distal tumors and intersphincteric resections in the Pontallier study [19].

Our study does not show any overall difference in urinary symptoms or sexual function. The study by Pontallier et al. [19] showed a trend towards better erectile function with a higher rate of sexual activity in the transanal group. This study suggests that the transanal approach allows better preservation of the pelvic nerves.

Our data suggest that functional results are comparable after transanal and laparoscopic TME, despite that we are still in the early phase of implementation of this technique. The transanal approach to TME is still developing into a standardized approach and may have enormous advantages over laparoscopic or open surgery. TaTME improves visualization of the surgical planes, which improves the quality of the surgical specimen and may reduce the need for conversion. We recently published short-term data on our first 80 patients, in which specimens were graded as complete or nearly complete in 97%. CRM positivity was seen in 2.5% of patients and the distal margin was free in all patients [2]. Since we first implemented transanal TME in 2012, the technique has evolved considerably. We initially worked with one team, starting transanally. We currently use a two-team approach, as advocated by Lacy and coworkers [21], wherein the splenic flexure is mobilized, followed by mobilization of the sigmoid and upper rectum by the abdominal team, while the transanal team simultaneously mobilizes the low and mid rectum [22]. Another significant change in protocol we introduced during the learning phase was the level of the proximal purse string suture. In our early experience, the purse string was often placed unnecessarily low. A better understanding of down-to-up anatomy led us to appreciate that low purse string sutures are often unnecessary, as the mesorectum frequently starts much higher than initially thought. Depending on the tumor location, the purse string is placed approximately four centimeters from the dentate linea, leaving ample space for a stapled side-to-end anastomosis.

Despite the shortcomings of TaTME as currently implemented, this approach to TME has afforded patients with low rectal cancer who have a narrow pelvic space, in which dissection and division of the rectum below the tumor has proven to be impossible, another alternative. Prior to the introduction of TaTME, these patients were often faced with an end colostomy because the procedure is generally converted to an intersphincteric dissection or even APR in order to allow dissection in the distal rectal region. TaTME has allowed patients who would previously only qualify for colostomy the option for a low anastomosis. Although TaTME may influence short-term functional results, the current data do not support this finding with comparable short-to-medium term outcomes between laparoscopic and transanal TME.

The comparison of consecutive groups in the current study, which resulted in differences in length of follow-up and small sample size, could be interpreted as a flaw in the study design. Unfortunately, the TaTME approach was employed at the end of the study period and became the preferred approach to TME, which did not allow comparison of the two techniques. Although it is generally accepted that functional results will stabilize over a period of time, this factor could have influenced the results.

All patients in the TaTME group were operated by a single surgeon in one hospital. Outcomes of this patient group could therefore have been influenced by a learning curve, as some included patients were one of the first in this hospital who underwent TaTME. Despite this possible learning curve, comparable functional and quality of life outcomes were measured in this study.

Another study limitation is the type of anastomosis employed since previous literature has proven that type of anastomosis could be of influence on functional outcome [23, 24]. Patients in the LAR group had a side-to-end anastomosis, while patients in the TaTME group had an end-to-end anastomosis. However, this difference is not expected to influence the results of our study since the type of anastomosis will only effect short-term functional outcomes and stabilizes after six months as results described in current data [23]. Although patient age also differed between the groups, we are not aware of any relation between age and function. Other patient characteristics known to be of influence on functional outcome such as tumor height and type of neoadjuvant were equally represented in both groups.

## Conclusion

This study shows that anal dysfunction may occur after both laparoscopic and transanal TME, comparable to the published literature. However, due to limitations in this study, these results should be interpreted with caution and prospective randomized studies will be required to support these data.
